# Neuromodulation for pediatric disorders of consciousness: bridging the gap between adult evidence and developmental reality

**DOI:** 10.3389/fneur.2026.1806510

**Published:** 2026-03-30

**Authors:** Yang Zhang, Xiaoke Zhao

**Affiliations:** Department of Rehabilitation, Children’s Hospital of Nanjing Medical University, Nanjing, China

**Keywords:** developing brain, neuromodulation, neuroplasticity, pediatric disorders of consciousness, precision medicine, rTMS, tDCS

## Abstract

Pediatric disorders of consciousness (pDoC) impose a disproportionate lifelong burden on patients and families, yet therapeutic options remain discouragingly limited. While neuromodulation has emerged as a paradigm-shifting intervention for adults, its translation to the pediatric population is hindered by a lack of specific evidence and the physiological complexities of the developing brain. In this review, we bridge the gap between adult-derived findings and pediatric reality. We synthesize current evidence regarding non-invasive (tDCS, rTMS) and invasive (DBS) techniques, with a specific focus on the anatomical (e.g., skull conductivity) and physiological (e.g., synaptic plasticity) disparities that invalidate the “miniature adult” approach. Furthermore, we address the unique ethical dilemmas and safety challenges inherent to pediatric neurostimulation. Finally, we discuss the critical need for precision medicine, advocating for the integration of multimodal neuroimaging and computational head modeling to develop individualized, age-appropriate stimulation protocols.

## 1 Introduction

Chronic disorders of consciousness (DoC) comprise a spectrum of severe neurological conditions characterized by prolonged impairments in arousal and awareness lasting at least 28 days, most commonly including unresponsive wakefulness syndrome (UWS) and the minimally conscious state (MCS) (1, 2). While the prevalence of pediatric DoC (pDoC) is lower than in adults—estimated at approximately 1–4 per 100,000 children—the etiological landscape and disease burden differ fundamentally (3). In adults, stroke and degenerative diseases predominate; in contrast, pediatric cases are largely associated with acquired brain injuries. A recent multicenter cohort study of 383 children highlighted this disparity, identifying traumatic brain injury (TBI, 36.8%) and anoxic–ischemic encephalopathy (12.3%) as major etiologies, whereas cerebrovascular accidents were rare (<0.3%) (4). This distinction is clinically relevant, as anoxic injuries in children are consistently associated with poorer prognostic outcomes than traumatic causes, with meta-analytic evidence suggesting that full recovery rates in severe cases may be as low as ~17% (4, 5).

Furthermore, the long-term trajectory of pDoC imposes a substantial multidimensional burden on family caregivers and generates considerable, sustained costs for public healthcare systems. Advances in critical care have increased survival rates among pediatric patients, often resulting in prolonged survival with severe disability rather than early mortality, a phenomenon often referred to as the “survival-disability paradox” (6, 7).

Current therapeutic strategies for pDoC remain limited, and no definitive disease-modifying treatments have been established (6). Pharmacological management remains largely empirical. For example, although amantadine is prescribed in approximately 45% of pediatric cases and is the only agent recommended by clinical guidelines (6, 8), evidence supporting its efficacy in children remains limited (9). Similarly, zolpidem has not demonstrated consistent long-term benefit in pediatric cohorts, with small-sample studies yielding heterogeneous results (10). Conventional rehabilitation strategies in this population often emphasize complication prevention, with limited evidence supporting interventions aimed at directly promoting neural repair.

Against this backdrop, neuromodulation has gained increasing attention as a strategy to modulate large-scale neural networks involved in arousal and awareness. Meta-analyses of randomized controlled trials indicate that repetitive transcranial magnetic stimulation (rTMS) produces moderate improvements compared with sham stimulation, including statistically significant gains in Coma Recovery Scale–Revised (CRS-R) scores, particularly in patients in an MCS (11–13). Although evidence for transcranial direct current stimulation (tDCS) remains comparatively limited (13), emerging pediatric-specific data are encouraging; a recent randomized trial reported that 5 Hz rTMS combined with rehabilitation was associated with improvements in both CRS-R and Glasgow Coma Scale (GCS) scores in children (14).

Despite these findings, the applicability of adult neuromodulation paradigms to pediatric populations remains uncertain (13, 15). Most current approaches in pDoC are extrapolated from adult studies (9), reflecting the implicit assumption that children can be treated as “miniature adults” (16). However, the pediatric brain is structurally and functionally distinct.

MRI-derived and computational head models indicate that children typically have thinner skulls and higher relative cerebrospinal fluid (CSF) volumes than adults, factors that can substantially alter the distribution and magnitude of induced electric fields (17, 18). Under commonly used adult stimulation parameters, modeling studies suggest that pediatric heads may experience elevated cortical electric field intensities, introducing a theoretical risk of excessive current density in developing tissue (18).

In addition, the developing brain undergoes dynamic processes of synaptic pruning and active myelination (19), such that external modulation during sensitive developmental windows may alter maturation trajectories and carry a risk of maladaptive plasticity (20). Although short-term safety profiles are generally acceptable, the long-term neurodevelopmental impact of repeated stimulation remains unclear, and standardized pediatric-specific protocols are lacking (21). Accordingly, this review synthesizes current evidence on non-invasive and invasive neuromodulation in pDoC. We argue that differences in cranial biophysics and neurodevelopmental processes in children substantially limit the direct applicability of adult-derived neuromodulation protocols. Consequently, the field must abandon the “miniature adult” premise and establish precision-guided frameworks as the new standard for pediatric neurostimulation.

## 2 Mechanistic rationale and developmental constraints

### 2.1 The neural mechanism of consciousness recovery

Contemporary models of consciousness recovery are commonly framed by the “mesocircuit” hypothesis, first systematically articulated by Schiff (22). The model proposes that arousal and awareness depend on the functional integrity of the forebrain cortico-striatal-thalamic-cortical (CSTC) loop (23). In DoC, diffuse or focal brain injury commonly leads to cortical deafferentation, which reduces striatal drive and increases inhibitory output from the globus pallidus internus (GPi) to the central thalamus (24). This “functional disfacilitation” of the central thalamus contributes to a widespread reduction in thalamocortical drive and fronto-parietal network activity, providing a network-level explanation for impaired behavioral responsiveness (23).

Neuromodulation strategies are intended to restore communication within this disrupted circuit and enhance large-scale network excitability. Non-invasive brain stimulation (NIBS), including rTMS and tDCS, typically targets cortical hubs such as the dorsolateral prefrontal cortex (DLPFC) or primary motor cortex (M1). Network-level and neurophysiological studies suggest that these interventions may exert a “top-down” modulatory effect, with induced activity propagating beyond the stimulation site to influence distributed corticothalamic pathways (15). In contrast, invasive approaches such as deep brain stimulation (DBS) deliver direct “bottom-up” input to thalamic nuclei (e.g., the centromedian–parafascicular complex), thereby bypassing excessive GPi-mediated inhibition and facilitating reactivation of thalamocortical arousal networks associated with behavioral responsiveness (25, 26). These mechanistic assumptions, however, are largely derived from adult neuroanatomy and network organization, raising important questions about their applicability to the developing brain.

### 2.2 Anatomical and physiological challenges in the developing brain

Translating adult-derived neuromodulation paradigms to pediatric patients is complicated by both biophysical differences that shape dose delivery and developmental factors that shape network responsiveness. Imaging-derived head models indicate that children typically have thinner skulls (e.g., ~2.9 mm vs. 3.9 mm in adults) and lower cranial impedance, along with a higher CSF-to-brain volume ratio (18, 27). Computational studies consistently show that these features can substantially alter electric field distributions (28). Under commonly used adult montages (e.g., 2 mA tDCS), modeling studies suggest that peak cortical electric fields in children may be up to ~twofold higher. However, such estimates depend heavily on specific modeling assumptions and age-related anatomical variability, warranting cautious interpretation (18, 29). Accordingly, modeling work has proposed that lower stimulation intensities (e.g., ~0.7 mA in some scenarios) may be required to approximate adult-like cortical field strengths (30).

**Figure 1:**
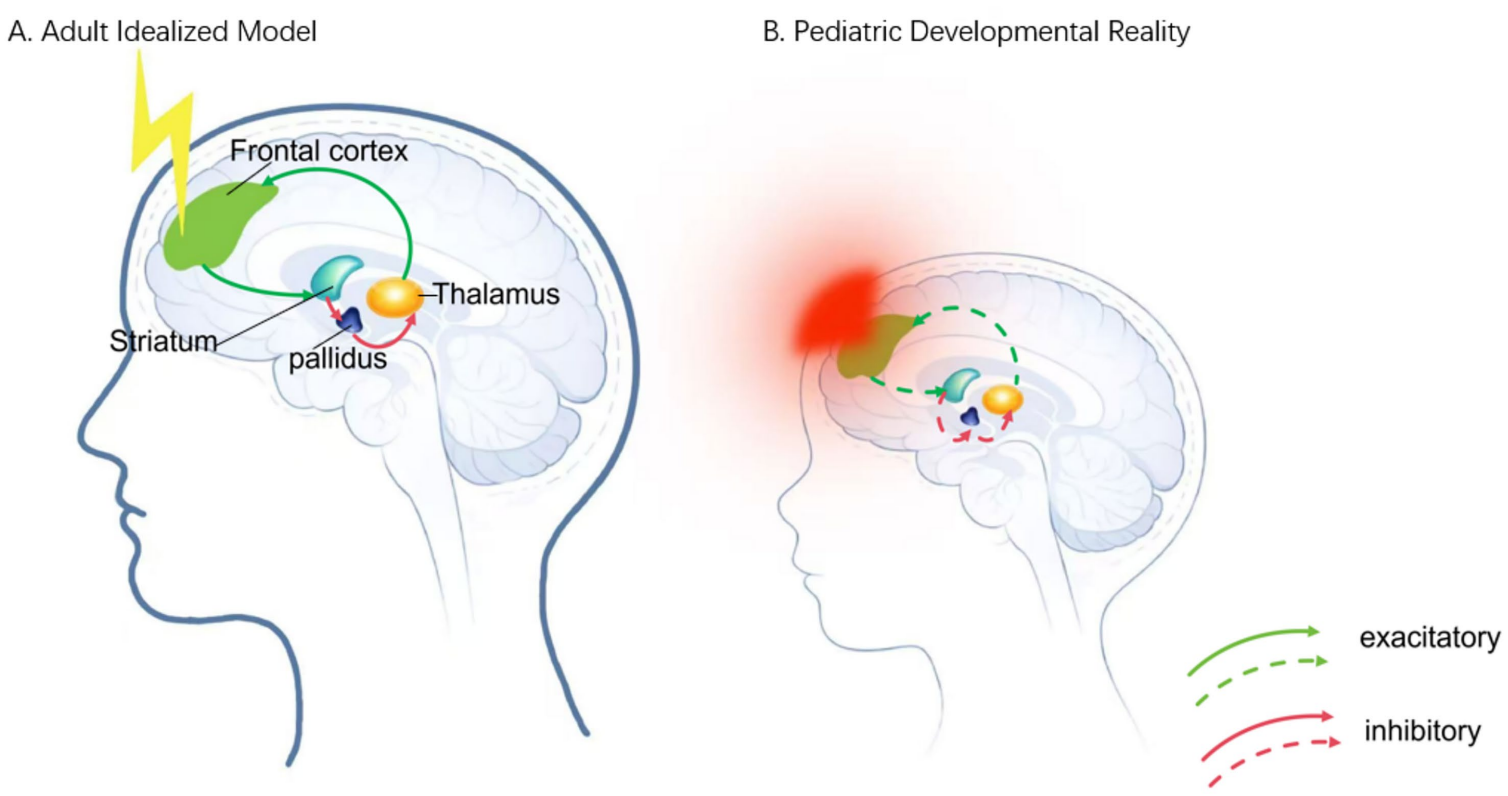
The mesocircuit hypothesis of consciousness recovery versus developmental barriers in the pediatric brain. **(A)** Adult idealized model. The mature brain is depicted as a stable CSTC loop. Neuromodulation (yellow lightning bolt), typically applied over the DLPFC, is hypothesized to enhance cortical drive to the striatum, which subsequently inhibits the GPi/SNr. This disinhibition of the central thalamus facilitates excitatory thalamocortical output to the frontal cortex (solid green arrows), thereby re-establishing a closed functional loop essential for consciousness recovery. **(B)** Pediatric developmental reality. In contrast, the developing brain presents anatomical and physiological constraints that challenge the “miniature adult” assumption. The thinner pediatric skull and lower tissue impedance facilitate increased electric field penetration, illustrated by diffuse red shading extending toward deep structures, which raises the theoretical risk of excessive current density or unintended stimulation of subcortical targets. Concurrently, ongoing synaptic pruning and immature network organization are represented by fragmented, dashed CSTC pathways, suggesting that the functional connectivity required for canonical neuromodulation may be structurally discontinuous or unstable during development. CSTC, cortico–striato–thalamo–cortical; DLPFC, dorsolateral prefrontal cortex; GPi, globus pallidus internus; SNr, substantia nigra pars reticulata. Not to scale.

Beyond biophysics, neurodevelopmental processes—particularly synaptic pruning and ongoing myelination—shape excitability and functional connectivity across development (31). As a result, canonical stimulation targets such as the DLPFC may exhibit different connectivity profiles across developmental stages (19, 32). Taken together, a uniform, adult-derived stimulation paradigm is unlikely to be appropriate for pDoC (6). Protocols that incorporate age, neurodevelopmental stage, and individualized head modeling may therefore be necessary to improve precision and safety (33) (Figure 1).

## 3 Non-invasive brain stimulation

### 3.1 tDCS

tDCS has gained increasing interest in pediatric neurorehabilitation, largely due to its portability, low cost, and generally favorable safety profile. Unlike rTMS, tDCS modulates resting membrane potentials rather than directly inducing action potentials; in most experimental contexts, anodal stimulation is associated with increased cortical excitability, whereas cathodal stimulation is associated with reduced excitability. In pDoC, the DLPFC is a commonly used target, reflecting its role within executive and fronto-parietal control networks.

Although efficacy data in pDoC remain limited, the safety and feasibility of tDCS in pediatric populations are supported by early-phase trials and broader pediatric safety studies. A Phase I dose-escalation study by Saleem et al. established a preliminary safety framework for pDoC by implementing a titrated dosing regimen (0.5–2.0 mA) based on head circumference to mitigate the risk of excessive current density (34). This profile is consistent with findings from systematic reviews and large-scale prospective studies encompassing more than 800 stimulation sessions, which reported no serious adverse events and only minor, transient side effects such as itching or tingling (35, 36).

Randomized controlled trials in children with hemiparesis (37) and neurodevelopmental disorders (e.g., attention-deficit/hyperactivity disorder and autism spectrum disorder) (38) further support the tolerability of tDCS in developing brains, including those with underlying structural pathology. However, while these studies suggest potential behavioral benefits in other pediatric domains, direct evidence of efficacy in pDoC remains sparse. Future research will likely need to move beyond single-session feasibility designs toward multi-session, individualized stimulation protocols to better assess the capacity of tDCS to induce sustained neuroplastic and functional changes in this population (39).

### 3.2 rTMS

rTMS operates via electromagnetic induction to generate intracranial currents sufficient to depolarize neurons and trigger action potentials, a mechanism distinct from the subthreshold membrane modulation characteristic of tDCS (40). In adult DoC, clinical guidelines and controlled studies support the use of high-frequency rTMS (typically 10–20 Hz) applied over the primary motor cortex (M1) or DLPFC to enhance cortical excitability and promote behavioral signs of consciousness recovery (2, 40).

Exploratory small-scale studies in pediatric acquired brain injury (ABI) have been reported; however, extrapolation to pDoC warrants heightened physiological monitoring and risk assessment. The pediatric brain exhibits relatively immature inhibitory networks and higher baseline cortical excitability compared with adults (41). When combined with the disrupted homeostatic mechanisms associated with severe brain injury, these features may increase the theoretical risk of seizure induction, the most serious adverse event associated with rTMS (36). Insights from the broader pediatric rTMS safety literature indicate that structurally malformed brains and anatomical lesions inherently lower the seizure threshold by altering local network excitability.

To ensure safe implementation in pDoC, clinicians must clearly distinguish absolute contraindications, such as uncontrolled clinical epilepsy or active status epilepticus, from risk factors that may be managed with appropriate precautions. Specifically, pharmacologically controlled epilepsy or the presence of interictal epileptiform discharges do not strictly preclude treatment if rigorous pre-stimulation EEG monitoring is employed (42). For such higher-risk pediatric patients, proconvulsant risks can be effectively mitigated by reducing stimulation intensity or utilizing low-frequency (≤1 Hz) inhibitory rTMS over hyper-excitable regions, which offers a significantly wider safety margin than canonical high-frequency paradigms (36, 42). Accordingly, existing safety guidance emphasizes rigorous risk stratification and physiological monitoring, including pre-stimulation electroencephalography (EEG) screening to identify active epileptiform activity, as part of standard practice for rTMS administration (43). Current ethical and safety consensus generally supports restricting rTMS in pDoC to specialized centers equipped with comprehensive neurophysiological monitoring and seizure management capabilities.

### 3.3 Emerging modalities: tACS and transcutaneous auricular vagus nerve stimulation (taVNS)

Given the variable efficacy and safety constraints associated with tDCS and rTMS in pediatric populations, alternative non-invasive neuromodulation approaches are being explored (44, 45). Transcranial alternating current stimulation (tACS) represents a mechanism-driven strategy designed to modulate endogenous neural oscillations and potentially restore disrupted functional connectivity through spike-timing–dependent plasticity (46–48). While tACS has demonstrated the capacity to influence cognitive processes in healthy adults and limited pediatric cohorts (e.g., attention-deficit/hyperactivity disorder and working memory paradigms) (49, 50), direct evidence for its application in pDoC remains sparse (51).

taVNS offers a complementary “bottom-up” approach (45). By stimulating the auricular branch of the vagus nerve, taVNS engages the nucleus of the solitary tract, with downstream modulation of the ascending reticular activating system and locus coeruleus–norepinephrine pathways implicated in arousal regulation (52). Because taVNS acts through peripheral vagal afferents rather than inducing currents in the cortical parenchyma, it offers a theoretical advantage for reducing seizure risk in vulnerable populations. In combination with evidence from human studies indicating that adverse effects are generally mild and transient, this feature supports a favorable safety profile for taVNS, particularly in pediatric populations (53). Together, its non-invasive and portable design and its tolerability profile position taVNS as a promising candidate for further systematic investigation in pDoC (54). Importantly, as current support for both tACS and taVNS relies predominantly on indirect evidence, these signals must be strictly viewed as hypothesis-generating rather than proof of efficacy. Consequently, establishing their therapeutic value in pDoC remains a future research goal that necessitates rigorous, sham-controlled trial requirements.

## 4 Invasive techniques

### 4.1 DBS and vagus nerve stimulation (VNS)

Invasive neuromodulation approaches, while enabling direct modulation of deep neural structures, remain largely investigational in pediatric populations, reflecting a limited evidence base and substantial ethical and surgical considerations (2). DBS targets key nodes of the arousal network, most commonly the central thalamus or the centromedian–parafascicular (CM–Pf) complex, to modulate activity within the downregulated mesocircuit (55). Seminal work by Schiff and colleagues reported that bilateral central thalamic DBS in an adult with severe TBI was associated with improvements in functional communication and limb control (55). However, pediatric applications remain largely limited to rare published case reports, often occurring in the context of treatment for movement disorders such as dystonia rather than as a primary intervention for disorders of consciousness (56).

VNS, delivered via an implanted pulse generator connected to the left cervical vagus nerve, is an approved adjunctive therapy for drug-resistant epilepsy in pediatric patients (57). Extensive clinical experience in epilepsy supports its surgical safety and tolerability profile in children (58). However, evidence for its role in promoting recovery of consciousness remains limited and is largely derived from isolated adult case reports (45).

Spinal cord stimulation (SCS) has also been explored as a means of enhancing cerebral perfusion and arousal, particularly in regional cohorts (59). Nevertheless, it lacks high-level evidence in Western populations and remains largely unstudied in pediatric disorders of consciousness (59).

### 4.2 Ethical and practical barriers in pediatrics

The broader implementation of invasive neuromodulation in pDoC is constrained by ethical and physiological challenges. From an ethical perspective, the inability of pediatric patients to provide informed consent necessitates proxy decision-making by caregivers, who must weigh the risks of elective neurosurgical intervention against uncertain therapeutic benefit, often in the absence of reliable prognostic markers (60).

From a physiological and technical standpoint, somatic growth introduces challenges that are uncommon in adult populations. As children mature, implanted leads and extensions connecting neural targets to chest-mounted generators may be subject to migration, tension, or fracture, increasing the likelihood of surgical revision and cumulative risks of infection and hardware failure. Longitudinal data from pediatric dystonia cohorts highlight these real-world risks, demonstrating hardware complication rates of 15 to 25%, surgical revision rates exceeding 20%, and infection rates of 5 to 10% (61). In addition, the long-term neurodevelopmental consequences of chronic deep brain or nerve stimulation remain poorly characterized, raising concerns regarding potential effects on cognitive, behavioral, and network maturation processes (62).

Accordingly, current expert consensus generally supports consideration of invasive neuromodulation primarily in highly selected pediatric cases, typically after non-invasive options have been explored, rather than as a frontline therapeutic strategy (2).

## 5 Discussion and future directions

### 5.1 Limitations of current evidence

A comprehensive summary of key empirical studies is provided in Table 1. As illustrated, the evidence base is evolving from early Phase I safety protocols to more rigorous study designs. Notably, recent advancements include the first Class I evidence for rTMS efficacy in pediatric TBI (14) and the successful implementation of remotely supervised, home-based tDCS protocols (63). These studies not only represent methodological maturation, but also signal an early shift toward etiology-specific and individualized stimulation strategies. However, these promising short-term gains are primarily limited to TBI cohorts; their long-term durability, functional significance, and generalizability to other etiologies remain unestablished. While the therapeutic potential of neuromodulation in pDoC is evident, the current evidence base remains limited, predominantly comprising feasibility studies and case series, with scant high-level RCT evidence. A recent scoping review revealed that 43.2% of pediatric studies were single case reports, with only 3.8% classified as clinical trials (64). Furthermore, meta-analyses indicate that available randomized controlled trials (RCTs) are often plagued by small sample sizes and a high risk of bias, precluding definitive conclusions regarding efficacy (13).

**Table 1:** Summary of key empirical studies investigating neuromodulation in pediatric brain injury and disorders of consciousness.

Author/year (PMID)	Modality	Study design	Population	Intervention protocol	Key findings, safety, and evidence level
Zheng et al. (14) 41159198	rTMS	RCT (multi-center)	*N* = 98 children (≥2 y) with DoC post-TBI	5 Hz rTMS @ 80% RMT to left DLPFC; 1,000 pulses/session, once daily × 3 weeks; + standard rehab	Efficacy: Significant improvement in CRS-R and GCS scores vs. sham (plus reduced serum NSE). Safety: No seizures or serious adverse events. Evidence: Class I (moderate-quality RCT)—first robust trial showing rTMS can safely promote recovery in pediatric TBI-DoC.
Stein et al. (63) 40563733	tDCS	Pilot Trial (home-based)	*N* = 10 children with ABI (ages ~10–15)	Remotely-supervised tDCS at home; 10 sessions (20 min each) over 2 weeks; dose 1 mA (*n* = 5) vs. 2 mA (*n* = 5) to bilateral DLPFC; concurrent online cognitive training	Feasibility: High at-home adherence; all families could self-administer with remote monitoring. Safety: No serious AEs over 100 sessions; 1 child struggled at first but tolerated tDCS after gradual ramp-up. Evidence: Pilot (open-label) evidence supporting scalability of tele-neuromodulation for pediatric rehab.
Stein et al. (73) 40714689	tDCS	Cross-over Study (mechanistic)	*N* = 15 pediatric ABI (mean 12.7 y) + 15 healthy controls	Single-session tDCS (20 min, 1 mA) to left DLPFC or left inferior frontal gyrus vs. sham, during gamified attention task; each child received all three conditions in random order	Mechanistic outcome: No overall immediate behavioral gain from one tDCS session vs. sham, but marked inter-individual variability. Children with certain baseline EEG connectivity patterns or higher modeled E-field in cortex showed greater attention improvements. Safety: No serious adverse effects noted. Evidence: Exploratory mechanistic study – underscores need for personalized dosing (E-field modeling) and possibly multi-session stimulation for efficacy.
Curatola et al. (74) 37165387	tDCS	Case Series (novel combo)	*N* = 3 children in chronic UWS post-cardiac arrest (anoxic injury)	Anodal tDCS + hr-NGF: 20 min tDCS sessions (current ramped to 2 mA) paired with intranasal NGF (50 μg/kg per dose); two treatment cycles one month apart	Efficacy: All 3 showed improved EEG background and power spectra, with clinical signs of heightened arousal (e.g., new voluntary responses) after combined treatment. Safety: No side effects reported. Evidence: Level IV (case series)—suggests a potential *synergistic* effect of combining neuromodulation with neurotrophic therapy in severe anoxic DoC, but requires further validation.
Ryan et al. (75) 36624962	tDCS	Case Report	*N* = 1 adolescent (15 y) with severe TBI (MCS)	Anodal tDCS (20 min, 1 mA) over motor cortex before intensive physiotherapy sessions; 16 sessions over 4 weeks (inpatient rehab setting)	Outcome: After 4 weeks, patient improved from wheelchair-bound to walking with a walker; Gross Motor Function Measure 33 percentage points. Gains maintained at 3 months. Safety: tDCS well-tolerated (mild scalp itch during stimulation). Evidence: Single-case (Level IV)—cannot prove causality, but demonstrates feasibility of integrating tDCS into pediatric neurorehab with clinically notable improvements.
Saleem et al. (34) 31401607	tDCS	Phase I Trial (dose-finding)	*N* = 10 children (6–16 y) with prolonged DoC (UWS/MCS)	Anodal tDCS over left DLPFC; 20 min session. Dose escalated by head size: 0.5 mA (small heads) up to 2.0 mA (largest heads), per safety protocol. Each child received up to 3 sessions if tolerated.	Safety: 100% session tolerability; no serious adverse events. The head-circumference–based dosing successfully prevented excessive current density. Evidence: Foundational safety study (Class I for safety)—established age/size-appropriate tDCS dosing guidelines for pDoC. Efficacy not assessed (not powered for clinical outcomes).

ABI, acquired brain injury; AE, adverse event; CRS-R, Coma Recovery Scale–Revised; DLPFC, dorsolateral prefrontal cortex; DoC, disorders of consciousness; hr-NGF, human recombinant nerve growth factor; MCS, minimally conscious state; RMT, resting motor threshold; SAE, serious adverse event; TBI, traumatic brain injury; tDCS, transcranial direct current stimulation; rTMS, repetitive transcranial magnetic stimulation; taVNS, transcutaneous auricular vagus nerve stimulation; UWS, unresponsive wakefulness syndrome. Evidence-level classifications: Class I indicates high-quality, randomized controlled trials (RCTs) with low risk of bias; Level IV indicates uncontrolled studies, including case series and single case reports.

A major confounding factor hindering large-scale RCTs is the profound heterogeneity of the pDoC population. Unlike adult cohorts often dominated by stroke or TBI, pediatric cohorts exhibit a distinct etiological landscape characterized by a significant prevalence of infections (37.1%) and severe anoxic-ischemic injuries (12.3%), alongside metabolic disorders (4). This variability implies that a stimulation protocol effective for a TBI patient may be ineffective in an anoxic patient. The latter often suffers from more widespread structural damage to the mesocircuit, rendering the neural substrate less responsive to modulation (65). Given its profound influence on residual network viability and reasonable outcome expectations, etiology warrants consideration as a primary stratification variable. Consequently, the limited availability of etiology-specific data constitutes a significant knowledge deficit that future precision-guided trials should strive to address.

### 5.2 Toward precision-guided neuromodulation

To address these limitations, the field is increasingly likely to benefit from a transition away from uniform stimulation paradigms toward precision-guided approaches (66). Future protocols may integrate multimodal neuroimaging, including diffusion tensor imaging and resting-state functional MRI, together with high-density electroencephalography, to identify residual functional networks and viable neural substrates prior to intervention. Such strategies can reveal thalamocortical connectivity that may not be apparent on structural imaging alone (67).

Neuronavigation systems may further support individualized targeting by aligning stimulation sites with patient-specific cortical regions that are functionally coupled to thalamic or fronto-parietal networks, with the potential to improve targeting accuracy compared to generic scalp-based coordinates (68, 69). In parallel, computational head modeling based on individual MRI data represents an important tool for estimating electric field distributions and guiding dose selection, helping to account for developmental differences in skull thickness and CSF volume that can influence cortical current density (18). However, as a standardized pediatric workflow is not yet established, these resource-intensive methodologies await broader clinical applicability and are best viewed as research priorities rather than immediate clinical standards.

### 5.3 Home-based and remote-guided intervention

Given the chronic trajectory of pDoC, which often extends over months to years, the future of rehabilitation is likely to extend beyond the acute hospital setting (62). Portable modalities such as tDCS and taVNS have demonstrated safety and feasibility in home-based applications within pediatric populations, including children with cerebral palsy and neurodevelopmental disorders (70).

The integration of remote supervision and digital monitoring platforms may enhance treatment adherence by reducing logistical barriers associated with repeated in-clinic visits (70). This model also facilitates the implementation of longer-term and higher-frequency stimulation protocols, which may be necessary to induce sustained neuroplastic changes in the developing brain compared with intermittent, clinic-based interventions (71). In addition, home-based delivery has the potential to reduce the socioeconomic burden on families and healthcare systems by improving accessibility to ongoing rehabilitation services for children with severe and persistent impairments (72).

## 6 Conclusion

Neuromodulation represents a developing and potentially valuable component of the therapeutic landscape for pediatric disorders of consciousness. This review highlights that the translation of adult-derived evidence to pediatric practice is constrained not only by differences in scale, but by fundamental distinctions in cranial biophysics, neurodevelopmental dynamics, and network organization. The assumption that children can be treated as “miniature adults” is therefore insufficient to guide stimulation strategies in this population.

Progress in this field is likely to depend on a shift toward developmentally informed, precision-guided approaches that integrate multimodal neuroimaging, computational head modeling, and neurophysiological monitoring to define age-appropriate and biologically grounded stimulation parameters. In parallel, the chronic trajectory of pDoC suggests that scalable and portable neuromodulation modalities, supported by remote supervision, may play an increasingly important role in long-term rehabilitation.

Together, these strategies provide a framework for advancing neuromodulation research and clinical practice in a manner that balances innovation with safety, methodological rigor, and the unique biological and ethical considerations inherent to the developing brain.
